# Physiological Effects of Dietary Amino Acids on Gut Health and Functions of Swine

**DOI:** 10.3389/fvets.2019.00169

**Published:** 2019-06-11

**Authors:** Zhongyue Yang, Shengfa F. Liao

**Affiliations:** Department of Animal and Dairy Sciences, Mississippi State University, Starkville, MS, United States

**Keywords:** amino acid, gastrointestinal tract, gut health, gut function, feeding strategy, pig

## Abstract

Gut health has significant implications for swine overall health status and nutrient utilization, due to its various functions including digestion and absorption of nutrients, secretion of mucins and immunoglobulins, and selective barrier protection against harmful antigens and pathogens. Both the basic anatomical structure of the gut (such as epithelial cells) and its luminal microbiota play important roles for maintaining gut health and functions. The interactions between epithelial cells and luminal microbiota have significant impact on host nutrition and health through the metabolism of dietary components. Amino acids, which are major nutrients for pigs, are not only obligatory for maintaining the intestinal mucosal mass and integrity, but also for supporting the growth of microorganisms in the gut. Dietary amino acids are the major fuel of the small intestinal mucosa. Particularly, glutamate, glutamine, and aspartate are the major oxidative fuel of the intestine. Emerging evidence shows that arginine activates the mTOR signaling pathway in the small intestine. Utilization of glycine by the small intestinal mucosa to synthesize glutathione is a very important physiological pathway, and the role of glycine as a powerful cytoprotectant has also been recognized. The major end products of methionine and cysteine metabolism are glutathione, homocysteine and taurine, which play important roles in the intestinal immune and anti-oxidative responses. Threonine is highly utilized by the gut and is particularly important for mucin synthesis and maintenance of gut barrier integrity. Moreover, either a deficiency or an excess of dietary threonine can reduce the synthesis of intestinal mucosal proteins and mucins in young pigs. Various new functions of amino acids on gut health and functions have been discovered in recent years. Thus, this review is to provide some up-to-date knowledge for industry application of dietary amino acids in order to enhance swine gut health and functions, and also it is to provide a comprehensive reference for further scientific research in this regard.

## Introduction

The ultimate goal of swine production is to convert various feedstuffs into edible pork for human consumption. Thus, enhancing the efficiency of the bio-transformation of feed mass into swine body mass, especially, the lean mass, is the bottom line for improving the profitability, as well as the sustainability, of swine production (1, 2). During the course of this bio-transformation by the pig, two initial steps after feed ingestion are feed digestion and nutrient absorption, which mainly take place in the gastrointestinal tract (GI tract or GIT), also known as gut, of the pig. Besides feed digestion and nutrient absorption, pig gut also encompasses a number of other physiological and biochemical features including intermediary nutrient metabolism and energy generation. In addition, the gut is also the largest immune organ in the body with defense mechanisms consisting of barrier function and mucosal immunity (2, 3).

Like other monogastric animals, the GIT of pig harbors several 100 microbial species, contains over 20 hormones, digests and absorbs a vast majority of nutrients, and accounts for roughly 20% of body energy expenditure (3, 4). Thus, both the tissue of the GIT wall and the luminal microbiota play essential roles to maintain a healthy gut and gut functions. The interactions between gut physiology and gut functions occur among the complex features of epithelial mucosa, luminal microbiota, and dietary nutrients (5). Indisputably, a healthy gut in swine is crucial to the overall nutrient metabolism, physiological activities, body wellbeing, and production efficiency at every stage of pig's life (5). Any harmful challenges on gut health can negatively impact swine utilization of dietary nutrients, compromise the whole body health and, consequently, reduce their production performance (6).

Without doubt, a healthy gut is considered as a foundation for swine production. From this regard, the maintenance or enhancement of gut health is essential not only for animal welfare but also for the business profitability of swine production. In the industry practice, formulating ideal diets to enhance the nutritional effects on gut health has been fast becoming a reality (3, 7). For this reason, the primary goal of this review is to summarize some up-to-date knowledge concerning the effects of dietary amino acids (AAs) on swine gut health and functions for animal nutritionists and producers to consider when formulating swine diets, and also to provide a comprehensive reference for animal scientists to use when conducting further research (6) on the nutritional regulation of the gut development, integrity, and functions of swine. To systematically approach the literature, Google Scholar (https://scholar.google.com), the world's largest academic search engine, was employed with key words as “effect of amino acid on gut health of pig or swine” for acquiring those relevant, well-conducted studies, especially that over the last decade. During the literature search and selection process when a specific AA was identified, the name of this AA would have been used as a key word to conduct some further searching and selection.

## The Conception of Gut Health

It is generally considered that the primary function of the gut is to digest feed, absorb nutrients, and excrete waste to support animals' lives (4, 5). In order for a pig to well perform this primary function, a healthy gut is without doubt a prerequisite condition before anything else (8). That being said, in the scientific community, what kind of gut is a healthy gut is not an easy question to answer. In the literature, there are many morphological and functional criteria that have been used to describe gut health (5, 7), but there is no single simple definition that has been widely accepted without further questions (2, 9).

### Gut Health From Morphology Perspective

Similar to other monogastrics, the GIT of swine consists of five major anatomic sections that are mouth, esophagus, stomach, small intestine, and large intestine. The small intestine, further consisting of duodenum, jejunum and ileum, is a key section that plays a central role for the physiological, biochemical, and immunological functions of the GIT (4). For this reason, most researches studying the gut health of pigs refer to small intestine as GIT or gut of pigs (7). With the same justification, the primary attention of this review has also been given to the health and functions of the small intestine of pigs, especially, the young pigs.

The integrity of anatomical structure is the foundation for the normal functions of the gut. From a morphology perspective, gut structure varies along the GI location, which reflects the functional requirements for digestion and absorption of nutrients, as well as for microbial and chemical defenses (10). The epithelia of small intestine in general consist of a single layer of tall columnar cells called epithelial cells that further include five major types: absorptive enterocytes (the primary ones), Paneth cells (secreting antimicrobial substances), goblet cells (secreting mucins), enteroendocrine cells (secreting GI hormones), and miscrofold or M cells (presenting antigens to the underlying lymphoid cells) (7, 11, 12). All these cells are connected mainly by tight junctions, gap junctions, and desmosomes (7, 11). These connected cells are structurally arranged as villi and crypts. The villi are finger-like projections into the intestinal lumen while the crypts are invaginations between the villi (4).

The surface of each absorptive enterocyte on the villi further has many small projections called microvilli, and all microvilli collectively form an expanded apical surface called brush border. Besides expanding the surface for nutrient absorption, the plasma membrane of the microvilli embeds various enzymes that can help to complete the final stages of nutrient digestion (13). The crypts of the epithelia lie within a lamina propria which is rich in lymphocytes, eosinophils, and plasma cells. The epithelial cells on the villi are continuously replaced by the proliferation of the cells in the crypts and this process is called epithelium turnover. Basically, the cells in the crypts continuously maturate and migrate up to the villi, and after 3–6 days these cells will end up with apoptosis or exfoliation at the villus tips (7, 14). During the migration most of the immature cells differentiate and become mature enterocytes on the villi. Other cells become either enteroendocrine or goblet cells (15).

Villus height (V), crypt depth (C), and V:C ratio are three key morphological indicators of the overall health and functions of small intestine (6, 16). High V may result in a greater absorptive capability for the available nutrients (6). A low C indicates a decreased metabolic cost of epithelium turnover, as crypts function as a villus factory. Deeper crypts indicate faster tissue turnover for villus renewal, which may be needed in response to inflammation caused from pathogens or their toxins (17). A greater V:C ratio suggests increased nutrient absorption (16), decreased secretion of proinflammatory cytokines, reduced metabolic cost, and improved growth performance (17, 18).

On the top of the epithelial layer is a mucus layer that basically is of viscous secretion from goblet cells (7). The epithelial and mucus layers together constitute the first line of swine defense against the intestinal pathogenic challenges. This line of defense is immediately active and mostly non-specific (10). Because the mucus secreted from goblet cells acts as a physical barrier against foreign substances, the count of goblet cells is a good indicator of potentially higher mucus production and secretion to protect the large area of the epithelial surface (19).

The tight junctions, located in the top of the intercellular structure, are multi protein complexes consisting of transmembrane proteins, such as occludin, claudins, tricellulin, and junctional adhesion molecules (20, 21). Those proteins may interact with the cytosolic peripheral proteins, including zonula occludens protein-1 (ZO-1), ZO-2, and ZO-3 (22), to form a selective physical barrier or para-cellular permeability that prevents the diffusion of molecules with <4 Å diameter in size (11, 21). Thus, the epithelial tight junctions commonly form a strong barrier against the absorption of endotoxins from the colonic lumen into mesenteric circulation (21). Overall, the layer of epithelial cells, together with the mucus layer on top of it and the lamina propria beneath it, provide an optimal micro-environment for chemical digestion, selective permeability, and partly resistance against endotoxins and pathogens (15).

### Gut Health From Microbiota Perspective

Modern nutritional researches have learned that besides the normal anatomical structure, as overviewed above, an appropriate luminal microbiota is also an indispensable component for gut health and functions (2, 10). Pig gut is sterile at birth and then colonized by numerous microbes from the dam, feed and environment, starting with lactic acid bacteria, enterobacteria, and streptococci (23), followed by many species of obligate anaerobes (6). Later, the gut microbiota is established as a stable complex micro-ecosystem composed of approximately 10^14^ (or 10^6^-10^12^/g of GI content) microorganisms with most of them being bacteria or anaerobic bacteria (roughly 400 to 640 species, representing approximately 140 genera), which symbiose with the pig as host (3, 4, 24). In humans and monogastric animals, the numbers of bacteria increase from 10^4^ cells per gram of digesta in the stomach to 10^11^ cells per gram of digesta in the large intestine (25). In mammals, the gut microbiota is characterized by its high population density, wide diversity, and interaction complexity. In terms of diversity, the gut microbiota consists of a mixture of bacteria, yeasts, protozoa, and virus (4). It is widely believed that almost all of these microorganisms are beneficial to the host, but some of them can be harmful or opportunistically harmful to the health of the host (7).

The activities of gut microbiota have potential effects on host nutrition and health through the metabolism of dietary components and through the interaction with intestinal epithelial cells (25). Studies investigating how bacteria contribute to the development of host intestinal functions have revealed a surprisingly symbiotic relationship between bacteria and the host (10). The gut microbiota salvage energy from otherwise indigestible carbohydrates, and protect the host from pathogens by forming a front line of mucosal defense (26). The commensal microbiota can also direct some postnatal development of the intestine (10). As Stappenbeck et al. (27) reported, certain commensal bacteria may be necessary for the development of intestine to its full absorptive capacity. Some member species in the GI bacterial community also exhibit anti-inflammatory effects on the mucosa. Neish (28) described a mechanism by which some bacterial proteins act as inhibitors of the NF-κB inflammation activation pathway. Overall, the bacterial cells outnumber animal (host) cells by a factor of 10 and have a profound influence on the nutritional, physiological, and immunological processes of the host.

The microbiota in the gut is now regarded as a multicellular, multifunctional organ whose genomes have genetic codes responsible for providing metabolic functions that host has not yet acquired or evolved in its own genome (10). In this regard, a focus on gut health should not forget to support the microbial ecosystem in the gut, to maintain its equilibrium, or to adjust this ecosystem when it is unbalanced (2, 5).

### Gut Health From Immunity and Anti-oxidation Perspectives

A well-developed immune system with optimal immune responsiveness is very important for animal's overall health and productive performance (7). Swine gut immune system consists of three lines of defense: barriers, innate immunity, and acquired or adaptive immunity that work together to protect the GIT and the whole body from diseases (29). The resident immune cells (e.g., T, B, and plasma cells), lymphocytes (for acquired immunity), macrophages and cytokines (pro-inflammatory or anti-inflammatory), dendritic cells (for innate immunity), and the related lymphoid tissue associated with GIT all together constitute this largest immune organ in the body (5, 30). Indeed, GIT is a home to more than 70% of all the host's immune cells (30).

The secretion of hydrochloric acid (HCl) by the stomach plays an important role in protecting animal body against the expansion of pathogens that were ingested with feed or water (31). In addition to the acid bath in stomach that causes a several log reduction in microbial counts, there are three component barriers: mucous and mucins, antimicrobial proteins, and secretory IgA. In pigs, the barrier system normally eliminates 99.9% of all infections and that is why it is also known as a “kill zone” level (29). Nonetheless, to any violation in the barrier function, or in the event of pathogenic, antigenic or allergenic challenge, the gut immune system must rapidly and strongly respond to mobilize its innate and adaptive immunities, which is critical in preventing the systemic spread of infection and inflammation (5, 32). The intestinal immune responses could lead to inflammatory responses and secretion of antibodies. As a critical innate immune process, inflammatory responses seek to contain an infection, activate adaptive immunity, repair damaged tissues and return to an immune homeostatic state. However, over inflammation costs more nutrients, and is associated with increased permeability that may lead to translocation of toxins, allergens, viruses, or even bacteria (5, 33). There is a plethora of interactions between immune cells, as well as with non-immune cells, which help to provide intestinal protection, tolerance and homeostasis (30), and all these related components work together in a coordinated way to prevent disease, maintain homeostasis, and maximize nutrient acquisition in the animal.

During the lifespan of the pig, various reactive oxygen and nitrogen species (ROS and RNS) are ordinarily produced from the aerobic cellular metabolism, which can accumulate under inflammation conditions (34). Some ROS, such as superoxide anion (O^2−^) and hydrogen peroxide (H_2_O_2_), are significant in the body that can damage cellular macromolecules, such as proteins, lipids, and DNA, and these damages can induce cellular oxidative stress and impair the integrity of mucosal epithelium, which would in turn cause serious problems with intestinal barrier functions. All these problems would lead animals to increased incidences of diseases (35).

Many practical efforts, such as hygiene, chemoprophylaxis, and vaccinations, have been made in prophylactic measures against swine infectious diseases. However, the immune responsiveness and anti-oxidation capacity cannot be maintained only by animal hygiene and vaccinations, but require adequate support from nutritional interventions. Furthermore, the immune alertness and reactivity of the gut can be modulated (oriented or improved) by nutritional components, such as dietary AAs (7). Moreover, the gut microbiota plays a prominent role in the development of gut immune system, particularly the adaptive immunity (2, 7).

Young pigs with early-weaned experience unavoidably face many health challenges or stressors resulted from numerous external and internal factors, such as the sudden change in diets with complex ingredients, anti-nutritional compounds, pathogens, potential toxins, and (or) antigenic molecules (36, 37). Despite a considerable amount of research conducted in the past, post-weaning problems, such as the well-known “growth check,” the occurrence of diarrhea, and the nervous signs, often followed by death, are still wide spread in the global swine industry (7). Even though the gut immune system in adult pigs is tightly regulated via a number of molecular mechanisms to prevent excessive activation and inflammation in response to internal stressors (5, 33), the gut immune system in young pigs is anatomically and functionally immature. It is evident that that more research directed toward overcoming the aforementioned problems associated with the immature gut of young pigs will be continued in the foreseeable future (6).

### Dietary Factors Influencing Gut Health and Functions

In swine, the overall gut health is influenced by many factors, such as the living environment, feeding strategies, microorganisms (including pathogens) and weaning practice, and is also mediated by some behavioral and psychological stresses (7, 38). Among all these factors, feeding strategies, especially the dietary nutrient components, play critical roles influencing pig gut health and functions (7, 14, 23). The ultimate interactions between gut tissue structure (especially, the epithelial cells) and gut microbiota (including their metabolites) have determinative influence on pig nutrition and whole body health through the metabolism of dietary components (5).

The major nutritional components in swine diets include carbohydrates (including fiber), proteins, AAs, and lipids, and all these components can have positive or negative impact upon gut health (6). It has been shown that the alteration of dietary protein quantity and (or) quality can manipulate the gut structure and functions, as well as the diversity and functions of the gut microbiota (39). Excessive amounts of dietary protein (more than required) can reach the lower GIT, and the fermentation of it can result in the production of various potentially toxic products (such as amines and NH_3_) and is often associated with the growth of potential pathogenic bacteria (such as *Clostridium perfringens*) and the reduction of fecal counts of beneficial bifidobacteria, especially in the piglets reared under nutritional and environmental stresses (40).

It is also known that a certain amount of fiber has to be included in diet to maintain the normal physiological functions of swine gut. Taking advantage of the potential prebiotic effects of dietary fiber has been considered as an effective measure to promote gut health and, thereby, minimize the use of anti-microbial growth promoters (AGP) in pigs (41). Dietary inclusion of soluble non-starch polysaccharides can stimulate the growth of commensal microbes in the gut (42). The addition of insoluble fiber sources, such as the husks from cereals, could reduce the excretion of hemolytic *E. coli* and the incidence of diarrhea after weaning (39).

A large number of feed additives have been evaluated for their effects on swine gut health. Organic and inorganic acids have positive effects on swine gut development and health, and in turn on the whole body health and productivity (6). The positive effects of these acids were attributed to various factors including: (1) the anti-microbial activity of non-dissociated organic acids, (2) lowering digesta pH (particularly, in the stomach) and aiding protein digestion, (3) lowering stomach emptying rate, (4) stimulating pancreatic enzyme production and activities in the small intestine, and (5) providing nutrients that are preferred by intestinal tissue thereby enhancing mucosal integrity and function (6, 39).

Essential oils have been used as artificial flavorings and feed preservatives, and some of those essential oils have strong anti-microbial activities (43). Another class of feed additives, exogenous enzymes, have also been utilized in swine diets to improve the digestive function of the gut (44). Jiang et al. (45) reported that combination of essential oils and an enzyme blend effectively improved the ileal morpho-functional aspects, down-regulated its inflammatory reaction, and modulated the fecal microbiology of the weaned piglets.

Due to the global push to eliminate the usage of antibiotics as AGP for pigs, searching for novel alternatives to the in-feed antibiotics to support the industry for profitable and sustainable pork production are currently ongoing. Some in-feed probiotics have been considered by many nutritionists as an ideal alternative to antibiotics (2). For young piglets, probiotics are expected to deliver at least one of the following functions to the gut: (1) stimulating the development of a healthy microbiota that is predominated by beneficial bacteria, (2) preventing enteric pathogens from colonization, (3) increasing digestive capacity and lowering the luminal pH, (4) improving mucosal immunity, and/or (5) enhancing gut tissue maturation and integrity (39). Prebiotics, another class of feed additives, could also benefit the host in a manner similar to probiotics (46). Combining prebiotics with probiotics may increase the efficacy of probiotic effects on gut development and health in newly-weaned piglets (2).

Moreover, there are also many other feed additives, such as immunoglobulin, ω-3 fatty acids, yeast derived ß-glucans, phytochemicals, and zinc oxide, used in swine diets in order to endorse their positive impacts on gut health and functions (6, 23, 47, 48). However, it is not an intention of this review to cover in details all these feed additives, as well as the aforementioned dietary nutrients. Instead, this review is focused on the beneficial effects of AAs, a very important group of nutrients for pork production. It should be kept in mind that the purpose, or the primary purpose, of inclusion of dietary protein component for swine is to provide individual AAs for the pig to use. In this regard, more and more commercially available feed-grade crystalline free AAs, such as lysine (Lys), methionine (Met), threonine (Thr) and tryptophan (Trp), have been commonly and increasingly supplemented to swine diets in practice.

## Effects of Amino Acids on Gut Health and Functions

### Gut Protein and Amino Acid Metabolism

Gut digestion of dietary protein by the pig begins in the stomach through the biochemical actions with gastric HCl and proteases; however, there was no evidence indicating any absorption of AAs or peptides in the stomach (49, 50). The small intestinal digestion of protein comprises both luminal and mucosal phases. In the lumen, large protein molecules are broken down by the active proteases and peptidases to release oligopeptides as well as free AAs (51). Oligopeptides of more than three AA residues are further hydrolyzed extracellularly by brush border peptidases. It was estimated that the products of protein digestion in small intestinal lumen consists of approximately 20% free AAs and 80% tri- and di-peptides (50). The tri- and di-peptides are further hydrolyzed by both brush border and cytoplasmic peptidases into free AAs, or are absorbed intact and transported into the blood circulation (50, 51).

Comparing with the intact dietary protein, the supplemental crystalline free AAs are absorbed more rapidly and completely than the protein-bound AAs in pigs. However, this rapid absorption could cause a temporary surplus of free AAs and result in an imbalance of available AAs at the sites of protein synthesis. These rapidly absorbed free AAs may be oxidized too quickly for protein accretion (52), and the efficiency of dietary free AA utilization might be reduced when slowly digested protein compose a large portion in the diet (53).

Using dietary non-protein nitrogenous substances, such as urea, ammonium, mucins, as well as the enzymatic secretions and sloughed epithelial cells of the host, the microbiota in pig intestine can also synthesize AAs and proteins for incorporation into bacterial cells (25, 51). It was revealed that the small intestine is also the major site for the microbe-derived AA absorption, and some investigators suggested that the biosynthesis of AAs and proteins by the microbiota in the host GIT partake in the regulation of AA homeostasis of the host (25, 54). However, the intestinal microbiota do not make a significant contribution of AAs to the host since pig exhibits a negative nitrogen balance when fed an AA- or protein-free diet (25).

Some dietary proteins in swine small intestine may escape full enzymatic digestion and flow directly to the large intestine where microorganisms can ferment. The resulting products of the fermentation include mainly microbial proteins but also some small metabolites, such as ammonia, free AAs, urea, methane, and short-chain fatty acids. The amount of free AAs synthesized, however, represents <1% of the total hindgut fermentation products (50).

In terms of gut AA catabolism, there is a substantial breaking down by small-intestinal mucosal cells, as well as by the intestinal microbiota. The major pathways of microbial AA catabolism are deamination and decarboxylation, and the metabolites of the catabolism include ammonia, amines, phenoles, indoles, short- and branched-chain fatty acids, organic acids, and some gaseous compounds (25, 51). These AA metabolites form a highly complex reservoir in the gut, which has significant impact on the physiology of the gut epithelia (55). Some metabolites (e.g., butyrate and indole) are beneficial, while others (e.g., ammonia) exert deleterious effects on the epithelia. It should be pointed out that the gut microbiota can recycle these metabolites for synthesize microbial proteins when needed.

As discussed in section Dietary Factors Influencing Gut Health and Functions above, numerous nutritional factors can influence pig gut health and functions. The content and types of dietary AAs in the intestinal lumen are amongst these factors (14, 56). AAs are obligatory not only for maintaining intestinal mucosal mass and integrity, but also for supporting the growth of luminal microorganisms, and further impacting the health and functions of the gut. Although it was reported that some AA metabolites (e.g., hydrogen sulfide and nitrite) can be taken up by the intestinal cells from the extracellular medium (i.e., luminal content) and exert deleterious effects on intestinal mucosa (25), numerous studies have shown that several AAs, including essential AAs, such as Lys, Met and Thr, and non-essential AAs, such as arginine (Arg), glycine (Gly), cysteine (Cys), glutamate (Glu) and glutamine (Gln), play critical roles in maintaining or promoting gut health and gut functions (56, 57). Table 1 provides an overview of the research during the last decade concerning the effects of some important AAs (quantitatively) on the health and functions of pig gut. In the following sections of this paper, the current, updated knowledge in the literature regarding the roles of AAs in supporting pig gut health and functions are discussed in more details. When necessary, some desired data from other monogastric animals, such as chickens, are also included.

**Table 1:** Overview of the research during the last decade concerning the effects of amino acids on pig gut health and functions^a^.

**Amino acid(s)**	**Dietary concentrations^b^**	**Animals examined on**	**Major effects on gut health observed**	**References**
Amino acid blend (AAB)^c^	1.00% AAB vs. 0.99% alanine	Weaner pigs (24-day-old)	Improved the intestinal morphology, barrier function, and antioxidative capacity; reduced the diarrhea incidence	(58)
Arginine	0.4 vs. 0.0 g/kg; twice daily	Newborn piglets	Showed a beneficial effect on the intestinal barrier system by reducing the trans-epithelial permeability in early rotavirus enteritis	(59)
Arginine	1.0 vs. 0.0%	Weaner pigs (5.3 ± 0.13 kg)	Increased the epithelial villus height and the mucosal vascular endothelial growth factor (VEGF) level of the small intestine	(60)
Arginine	1.0, 0.5, vs. 0.0%	Weaner pigs (21-day-old)	Protecting and enhancing intestinal mucosal barrier function; maintaining intestinal integrity	(61)
Arginine	1.0 vs. 0.0%	Growing pigs (55 kg)	Ameliorated the intestinal abnormalities caused by mycotoxin	(62)
Arginine	1.6, 0.8, vs. 0.0%	Weaner pigs (8.7 ± 0.43 kg)	Suppressed the inflammatory cytokine expression	(63)
Glutamine; glutamine + glutamate	1.00 vs. 0.00%; 0.88 to 0.66 vs. 0.00%	Suckling and nursery Pigs (14– to 21-day-old)	Increased the jejunal villus height by glutamine; increased the jejunal crypt depth by glutamine + glutamate	(64)
Glutamate	1.0 vs. 0.0%	Weaner pigs (5.6 ± 0.51 kg)	Improved intestinal mucosa morphology	(65)
Glutamate	2.0 vs. 0.0%	Growing pigs (55 kg)	Alleviated the adverse effects of mycotoxins on gut structure	(66)
Monosodium glutamate	4.0, 2.0, 1.0, 0.5, vs. 0.0%	Weaner pigs (21-day-old)	Increased jejunal villus height, DNA content, and antioxidative capacity	(67)
Monosodium glutamate	3.0 vs. 0.0%	Growing pigs (25.0 ± 1.3 kg)	Detrimental effects on several physiological and inflammatory parameters measured in the proximal intestine, while exerting some beneficial effects on the distal intestine	(16)
Glutamine	4.4 vs. 0.0%	Weaner pigs (21-day-old)	Improved the intestinal barrier function	(68)
Sulfur amino acids^d^	4.20, 2.90 vs. 1.30 g/kg	Growing piglets (18.6 ± 0.7 kg)	Enhanced the whole-body immune status	(69)
Sulfur amino acids^e^	1.15, 0.94, 0.89, 0.76, vs. 0.65%	Weaner pigs (21-day-old)	Improved intestinal functions via affecting the mucosal antioxidant systems	(70)
Methionine	4.0 vs. 0.0 g/kg	Weaner pigs (21-day-old)	Improved intestinal integrity and oxidative status	(71)
Methionine	0.145 vs. 0.000%	Weaner pigs (7.2 ± 0.97 kg)	Enhanced the duodenum morphology in association with reducing oxidative stress; Improved glutathione production in the mucosa cells	(72)
Cysteine	0.61 vs. 0.00%	Weaner pigs (28-day-old)	Increased the synthesis of mucosal epithelial proteins, such as glutathione and mucin	(73)
N-acetyl cysteine	500 vs. 0 mg/kg	Weaner pigs (14- t0 25-day-old)	Possessing a constructive regulation on the changes of the gut redox status and microbiota in response to weaning stress	(74)
Taurine	0.1 vs. 0.0%	Weaner pigs (5.8 ± 0.58 kg)	Decreased the stimulation of immune response to lipopolysaccharide (LPS); Improved intestinal epithelial barrier function	(75)
Tryptophan	0.4, 0.2, vs. 0.0%	Weaner pigs (7.6 ± 0.04 kg)	Aaltered intestinal microbial composition and diversity; Improved intestinal mucosal barrier function	(76)
Tryptophan	0.2 vs. 0.0%	Weaner pigs (8.9 ± 0.20 kg)	Improved the intestinal development; inhibited intestinal aging	(77)
Tryptophan	0.75, 0.15, vs. 0.00%	Weaner pigs (8.3 ± 0.15 kg)	Negatively affected intestinal morphology and tight junction proteins	(78)
Branched-chain amino acids^f^	Leu (1.38 vs. 1.26%), Ile (0.80 vs 0.60%), Val (1.01 vs. 0.74%)	Weaner pigs (28-day-old)	Enhanced intestinal development, and intestinal expression of amino acid transporters	(79)
Leucine	1.4 vs. 0.0 g/kg	Suckling pigs (7-day-old)	Improved the intestinal development; enhanced the expression of leucine transporters in the jejunum	(80)
Lysine	130, 100, vs. 70%	Young piglets (21.3 ± 0.39 kg)	Enhanced the richness and evenness of the intestinal microbial community	(81)
Threonine	8.5, 7.5, 6.5, 5.8, vs. 5.3 g/kg	Weaner pigs (10–25 kg)	Increased the humoral antibody production and serum specific IgG concentrations	(82)

a In the text, some data on other species including chickens, mice and humans were used. In this table, however, only the studies on pigs are listed.

b For each study, the last concentration on the list was, in general, of the control group.

c The AAB included glutamate: glutamine: glycine: arginine: N-acetylcysteine at 5:2:2:1:0.5.

d Sulfur amino acids contain methionine + cysteine.

e The specific dietary methionine + cysteine concentrations were 0.83 + 0.32, 0.71 + 0.23, 0.53 + 0.36, 0.49 + 0.27, vs. 0.33 + 0.32, respectively.

f *Branched-chain amino acids, including leucine (Leu), isoleucine (Ile) and valine (Val), were added to meet the recommendations*.

### Amino Acid Effects on Gut Morphology Maintenance

Dietary AAs play critical roles in providing fuels for intestinal mucosa and, especially, Glu and Gln are major fuels for small intestine. Glutamate is commonly produced from Gln by glutaminase in the small intestine, and it could be a preferable fuel to Gln for enterocytes when the activity of glutaminase was low (56). The dominant role of Glu as an oxidative fuel may have therapeutic potential for improving the function of the infant gut, due to the high turnover rate of gut epithelial cells in infants (83). However, a recent study in growing pigs (16) showed that dietary supplementation of Glu (in a monosodium form) had detrimental effects on several physiological and inflammatory parameters measured in the proximal intestine, while exerting some beneficial effects on the distal intestine. For example, the dietary Glu supplementation increased the mRNA expression of pro-inflammatory cytokines including tumor necrosis factor α (*TNF*α), interleukin (IL)-1β (*IL-1*β), *IL-6, IL-8*, and *IL-10*, as well as the kinase ataxia telangiectasia mutated (*ATM*), in the proximal intestine (duodenum and jejunum), while inhibiting the expression of these pro-inflammatory factors in the distal intestine (ileum and colon) (16).

Similar to Glu, Gln can provide metabolic fuel for the rapidly dividing cells (particularly, the lymphocytes and enterocytes), as well as other epithelial cells of the intestines (17, 56, 84). Dietary Gln supplementation has positive effects on gut development via increasing villus height and V:C ratio, and reducing crypt depth, due to its metabolic fuel function for the gut (6, 17).

Dietary Arg supplementation can attenuate the degree of tissue damage in intestinal ischemia, promote intestinal mucosa healing (85), and reverse intestinal dysfunction (86). Sukhotnik et al. (87) reported in rats that oral Arg supplementation improved the duodenal, jejunal and ileal weights and mucosal cell proliferation, as well as restored the intestinal absorptive function after ischemia. The significant effect of Arg on the growth of GI mucosa in a variety of research animals may be attributed to its unique role over polyamine biosynthesis (88, 89). Notably, polyamines produced by the gut microbiota are associated with intestinal mucosal protection and epithelial cell migration (85). In rats, Hurt et al. (90) also demonstrated that diets supplemented with Arg and Glu helped the maintenance of intestinal tissue oxygenation and/or brush barrier function, and improved systemic nitrogen balance. Arginine is the substrate for nitric oxide (NO) synthesis. Studies showed that either inhibition or overproduction of NO had injurious effects on the guts of pigs and other animals (91, 92).

Sulfur-containing AAs, such as Met and Cys, are also beneficial for maintenance of gut morphology. Luminal microbes are responsible for the extensive catabolism of dietary Met in the gut (56). A study on nursery pigs showed that adding Met in drinking water can improve small intestinal morphology by increasing villous height. Methionine can reduce the bacteria fermentation via improving nutrient digestion and absorption and leaving less substrates for bacteria to use (93). Dietary Met for nursery pigs can enhance the morphology of duodenum in association with reducing oxidative stress and improving glutathione (GSH) production in the mucosa cells (72). Cysteine is extensively utilized by animal gut (56). Bauchart-Thevret et al. (73) concluded that the gut of weanling pigs utilizes 25% of the dietary Cys intake, and that synthesis of mucosal epithelial proteins, such as GSH and mucin, are a major non-oxidative metabolic fate for Cys.

Threonine, with a high utilization rate by the gut, is well involved in intestinal maintenance and functionality (94, 95). It has been reported that Thr is an important component of mucins (40% of the mucus glycoproteins) in the gut (96). Dietary Thr supply is critical for maintaining gut morphology and development because Thr plays a key role in mucin synthesis and barrier integrity maintenance (56, 96, 97). Either deficiency or excess of dietary Thr, however, has adverse effects on the synthesis of intestinal mucosal proteins and mucins in young pigs (96). Chen (98) and Min et al. (96) both reported a positive effect of adequate dietary Thr supplementation on chicken gut morphology, such as villus height, epithelial thickness, number of goblet cells, and crypt depth in three segments of small intestine. Especially, the increasing crypt depth in the Thr-supplemented chickens might provide more surface area for nutrient absorption by increasing enterocyte proliferation and intestinal mucin secretion (96). Most researchers [such as (17)] consider low crypt depth as an indicator of decreasing metabolic cost of intestinal epithelium turnover, while deeper crypts indicating faster epithelium turnover for renewal of the villus as needed in response to the inflammation from pathogens or their toxins. In this particular study, however, Min et al. (96) explained that the deeper crypts indicate increased enterocyte proliferation, increased villus surface area, increased mucin secretion (because goblet cells are mainly present in the crypts) and, therefore, better nutrient absorption.

### Amino Acid Effects on Gut Luminal Microbiota

Although the contributions of the *de novo* synthesized microbial AAs to the AA requirements of pigs are still not certain (25), luminal AAs do have a significant impact on the microflora in the small intestine. Glutamate can markedly change the composition of, and increase the diversity of, the intestinal microbial community by promoting the colonization of *Faecalibacterium prausnitzii* and *Roseburia* (99). In addition, it has also been reported in a pig model that the addition of dietary Glu can help to modify the intestinal microbial composition to prevent obesity (99).

The extensive catabolism of dietary Lys in the gut is taken care by the luminal bacteria rather than the enterocytes and, therefore, it is postulated that that dietary Lys restriction can affect gut microbiota (56). Yin et al. (81) firstly reported that Lys restriction enhanced the intestinal richness and evenness of microbial community. Moreover, using a bioinformatics software package, Yin et al. (81) predicted that the altered intestinal microbiota caused by Lys restriction might influence AA metabolism, membrane transport, endocrine system, carbohydrate metabolism, cellular signaling, replication and repair.

Threonine not only regulates the protein homeostasis in the body but also supports the growth of bacteria in the gut (100). Dong et al. (101) reported that dietary Thr supplementation to a low crude protein diet for laying hens recovered the bacteria diversity caused by the low dietary protein, and increased the abundance of potential beneficial bacteria. One of the explanations for the increased bacteria diversity in this study might be the up-regulation of mucin gene expression by supplementing Thr, because mucins cannot be digested in the small intestine and thereby can reach the cecum, acting as a substrate for saccharolytic bacteria.

Extensive bacteria fermentation of undigested feed components in ceca is responsible for detoxification of harmful substances and prevention of pathogen colonization (102). The major products from bacteria fermentation are short chain fatty acids (a.k.a. volatile fatty acids), which play a key role in maintaining gut health by lowering luminal pH and regulating the microbial composition, especially by stimulating the growth of beneficial bacteria (101). A study on nursery pigs showed that adding a liquid Met analog (an acid form) in drinking water tended to decrease the gastrointestinal pH and the concentrations of cecum acetic acid. However, the total number of lactic acid bacteria and *E. coli* in cecum was not affected (93). Thus, Kaewtapee et al. (93) concluded that this liquid Met analog might enhance nutrient digestion and absorption (due to the increased villus height) and, subsequently, the growth performance. Therefore, less substrates remained for bacteria to use, which results in less metabolites (volatile fatty acids) from the fermentation processes (93).

### Amino Acid Effects on Gut Immunological Functions

Studies have shown that some AAs are critical in the maintenance of the immune-physiological functions of the gut (103, 104). Arginine is a central intestinal metabolite, both as a constituent of protein synthesis and as a regulatory molecule limiting intestinal alterations and maintaining gut immune-physiological functions (85, 86, 89). A large number of studies in animals and human have identified the important role of Arg in intestinal immunity (56, 103). Corl et al. (59) reported that early in rotavirus enteritis, Arg has a beneficial effect on the intestinal barrier system of piglets by reducing trans-epithelial permeability via a mammalian target of rapamycin/p70^S6k^-independent mechanism. Another study on pigs showed that supplementing 1% Arg to a mycotoxin-contaminated feed ameliorated the intestinal abnormalities caused by mycotoxin (62). Also, Hurt et al. (90) demonstrated in rodents that diets supplemented with both Arg and Gln enhanced the immunity of gut mucosa and helped maintaining intestinal tissue oxygenation and/or brush barrier function.

Threonine is an essential component of mucus glycoproteins (approximately 40% of the protein) in GIT (96). Threonine supply is critical for maintaining gut immunological functions by participating in mucin synthesis to maintain gut barrier integrity (56, 96, 97). Moreover, Thr has been reported to be an AA with the highest concentration in the γ-globulins of rabbits, horses and humans. Also, the humoral antibody production and serum specific IgG concentrations were all increased in response to the increased intake of true ileal digestible Thr in young pigs (82). All these results indicate that Thr is very important for the protection of gut mucosal barrier and for the immune functions.

As sulfur-containing AAs, Met and Cys have been shown to be beneficial for animal immune system (105–107). Methionine serves as a methyl donor for several important processes, such as DNA methylation and polyamine synthesis (108), which are important for enhancing immune cell proliferation during immune challenge. Cysteine is needed to produce taurine, acting as an antioxidant, as well as a cell membrane stabilizer (108). Taurine is particularly abundant in leucocytes (109). During the immune system stimulation (ISS), utilization of Cys for the production of the compounds involved in immune response, such as taurine and GSH, is increased (108). Rakhshandeh et al. (110) reported that the immune system stimulation by injection of lipopolysaccharide reduced the ratio of whole-body nitrogen to sulfur balance indicating that the sulfur-containing AAs are preferentially preserved for the production of non-protein compounds, such as GSH, to enhance the whole body immune status. This implies that more Met and Cys are needed during the state of immune challenge.

### The Anti-inflammatory Effects of Amino Acids

Studies have shown that some AAs can alleviate intestinal inflammation. Using a mouse model, Chau et al. (111) reported that dietary Arg supplementation reduced the expression level of ileal transcript mRNA encoding interleukin-4 (IL-4), a key mediator of intestinal mastocytosis and macromolecular permeability. The data suggested that increasing bioavailable Arg ameliorates intestinal inflammation and pathology. It is likely that the altered activities of Arg-catabolizing enzyme families, arginases and NO synthase (NOS), contribute to ameliorating allergic inflammation. It can be postulated that the activity of arginase through conversion of Arg into ornithine enhances epithelial barrier function. Recent studies have showed that NOS activity can dampen inflammation through regulation of the myeloid and lymphoid cell activation (85). Nitric oxide produced by inducible NOS in inflammatory monocytes and dendritic cells can regulate inflammatory cytokine production, cell differentiation, and survival (85). Modulating the arginase- and NOS-mediated pathways through regulation of the bioavailability of L-Arg or its precursor L-Cit via oral supplementation can provide an efficient and practical strategy to dampen intestinal inflammation and pathology, and regulate the mucosal immunohomeostasis (85).

Ample evidence also demonstrated that Gly has efficacy as an anti-inflammatory and cytoprotective agent (112). While the mechanism responsible for the protective effects of Gly are unclear, it is likely to be multi-factorial involving direct effects on target cells, inhibition of Gly-gated chloride channels, and/or inhibition of inflammatory cell activation. Some studies indicated that Gly has a protective effect in mesenteric ischemia/reperfusion (IR) injury through the inhibition of apoptosis (113), while others have shown that Gly protection against intestinal IR injury is reached by a mechanism consistent with Gly uptake (114).

### The Anti-oxidative Functions of Amino Acids

All AAs are susceptible to oxidation, although their susceptibilities vary considerably (115). Methionine and Cys are the most susceptible to oxidation by ROS. Because of this, they can thus help to defend cells against oxidative stress. Therefore, Met and Cys are considered as endogenous antioxidants (116) functioning through an important antioxidant defense mechanism (117, 118). The anti-oxidative ability of endogenous Met can protect many proteins from oxidative damage. Methionine in the diets for nursery pigs can enhance the duodenum morphology in association with reducing oxidative stress and improving GSH production in mucosa cells (72).

Glutathione is a major cellular antioxidant that functions to detoxify intestinal oxidative stress and injury related to microbe-induced inflammation (73). Several AAs execute antioxidant functions through GSH. Three AAs are needed to synthesize GSH: Gly, Glu and Cys. Glutamine is easily converted to Glu to produce an antioxidant GSH. Therefore, dietary supplementation of Gln may have beneficial effects in reducing the symptoms of inflammatory disorders and may protect the gut against the damaging effect of oxidative stress (84).

### Gut Dysfunction Reverse and Detoxification by Amino Acids

Many studies showed that AAs could be used to alleviate the adverse effects of toxins and gut barrier dysfunction. Through a study with 15 growing pigs, Duan et al. (66) concluded that Glu may be useful as a nutritional regulating factor to alleviate the adverse effects of mycotoxins on gut structure (histology, morphology and barrier function) and growth performance, because dietary Glu supplementation partially counteracted the impairments induced by the mycotoxins in the mold-contaminated feed.

Glutamine is a unique nutrient for enterocytes, capable of dual signaling and augmenting the effects of growth factors that govern cellular proliferation and reconstruction after damage (119). Souba et al. (120) suggested that Gln is an important AA in humans for maintenance of gut structure, metabolism, and function especially during critical illness when the gut mucosal barrier is compromised. Kessel et al. (121) reported that enteral feeding of Gln suppressed the injury to the mucous membrane of the small intestine caused by lipopolysacharide endotoxemia in rat. A recent study conducted by Xue et al. (17) in broilers suggested that Gln improved intestinal architecture in the jejunum and ileum during the necrotic enteritis outbreak and recovery and consequently favors intestine structure (increasing villus height and decreasing crypt depth) and functions. In mice, dietary Gln supplementation can block ethanol-induced gut permeability, and protect colonic epithelial tight junctions and adherent junctions (21, 122). Hence, dietary Gln supplementation can maintain gut barrier function and prevent alcohol-induced gut barrier dysfunction (21, 84, 122).

Glycine is not only an essential substrate for synthesizing several important biomolecules (such as glucose and GSH), but is also utilized in the biochemical detoxification via conjugation of endogenous or xenobiotic toxins (123, 124). A study with a rat model suggested that local Gly perfusion diminished the ischemia-reperfusion injury in small intestinal mucosa, as indicated by the increased mucosal protein content, increased mucosal DNA content, and maintenance of mucosal glutaminase activity, during either the pre-ischemia phase or the pre-reperfusion phase (114). Lysine also serves as a partial antagonist of gut serotonin 5-HT4 receptors to reduce stress-related diarrhea as well as anxiety, and may modulate gut motor function (125). Cysteine can modulate local cytokine gene expression, suppress pro-inflammatory and chemotactic gene expression, and promote the expression of pro-apoptotic pathways, in addition to its known anti-oxidant and immunological effects, suggesting that dietary supplementation of Cys may support the recovery of gut mucosal homeostasis (126).

## Conclusions

Overall, AAs are beneficial for maintaining gut health in pigs, especially from the morphology and microbiota perspectives. As summarized above, some AAs provide fuel for the growth and proliferation of intestinal epithelial cells, and others offer nutrients to luminal microbiota for maintaining its diversity and functions. Moreover, the types and levels of dietary AAs can differently or similarly affect the gut structure and functions. Arginine, Gln, Met, and Thr could help with relieving the post-weaning stress of young pigs by improving the immunological functions, anti-inflammatory ability, or anti-oxidant capacity. Glutamate, Gln, and Gly can reverse gut dysfunction under disease conditions and help to reconstruct the gut structure after its damage. Threonine, Arg, Gln, Met and Cys are all beneficial to protecting gut barrier function and maintaining gut mucosal immunity. Furthermore, Glu, Lys, and Thr play important roles in supporting and affecting the growth of bacteria in the intestinal lumen. That being said, the complex mechanisms underlying AAs' effects on gut morphology and functions still warrant further investigation. Considering the global push to ban the usage of antibiotics as AGP for swine production, our current primary effort may be made to explore the specific effects of individual AAs on gut microbiota of young pigs.

## Author Contributions

SL contributed the conception and structure design to the paper. ZY organized the literature and wrote the first draft of the paper. Both authors contributed to manuscript revision and approved the final version for submission.

### Conflict of Interest Statement

The authors declare that the research was conducted in the absence of any commercial or financial relationships that could be construed as a potential conflict of interest.
